# Decimal fraction representations are not distinct from natural number representations – evidence from a combined eye-tracking and computational modeling approach

**DOI:** 10.3389/fnhum.2014.00172

**Published:** 2014-04-01

**Authors:** Stefan Huber, Elise Klein, Klaus Willmes, Hans-Christoph Nuerk, Korbinian Moeller

**Affiliations:** ^1^Knowledge Media Research Center, TuebingenGermany; ^2^Department of Psychology, Eberhard Karls University, TuebingenGermany; ^3^Section Neuropsychology, Department of Neurology, University Hospital, Rheinisch-Westfälische Technische Hochschule Aachen UniversityGermany

**Keywords:** number comparison, decimal fractions, compatibility effect, string length congruity effect, computational modeling, artificial neural network

## Abstract

Decimal fractions comply with the base-10 notational system of natural Arabic numbers. Nevertheless, recent research suggested that decimal fractions may be represented differently than natural numbers because two number processing effects (i.e., semantic interference and compatibility effects) differed in their size between decimal fractions and natural numbers. In the present study, we examined whether these differences indeed indicate that decimal fractions are represented differently from natural numbers. Therefore, we provided an alternative explanation for the semantic congruity effect, namely a string length congruity effect. Moreover, we suggest that the smaller compatibility effect for decimal fractions compared to natural numbers was driven by differences in processing strategy (sequential vs. parallel). To evaluate this claim, we manipulated the tenth and hundredth digits in a magnitude comparison task with participants’ eye movements recorded, while the unit digits remained identical. In addition, we evaluated whether our empirical findings could be simulated by an extended version of our computational model originally developed to simulate magnitude comparisons of two-digit natural numbers. In the eye-tracking study, we found evidence that participants processed decimal fractions more sequentially than natural numbers because of the identical leading digit. Importantly, our model was able to account for the smaller compatibility effect found for decimal fractions. Moreover, string length congruity was an alternative account for the prolonged reaction times for incongruent decimal pairs. Consequently, we suggest that representations of natural numbers and decimal fractions do not differ.

## INTRODUCTION

In recent years, there was increased research interest in the cognitive mechanisms underlying multi-digit number processing (see Nuerk et al., 2011 for a review). Nevertheless, while considerable progress has been accomplished in understanding the processing of natural multi-digit numbers and also fractions, the cognitive mechanisms involved when processing decimal fractions have largely been neglected so far. This is particularly noteworthy because decimal fractions – just like multi-digit natural numbers – comply with the general base-10 place-value structure of the Arabic number system: the numerical value of each individual digit in a multi-digit Arabic number is determined by its position within the respective digit string (i.e., units, 10, 100, etc.). Any number can thus be written as a linear combination of powers of 10, each weighted with one from the set of 10 symbols (i.e., the digits 0, 1, 2, 3, 4, 5, 6, 7, 8, 9). For instance, 639 can be expressed as 6 × 10^2^ + 3 × 10^1^ + 9 × 10^0^. Different from natural numbers, decimal fractions are also composed of weighted powers of 10 with integer exponents smaller than zero, separated from the components with integer exponents larger than or equal to zero by the so-called decimal point^1^ (e.g., 63.9 = 6 × 10^1^ + 3 × 10^0^ + 9 × 10^-^^1^). Decimal fractions may thus be considered just an extension of natural numbers.

Despite these structural similarities there are at least two important differences when individuals have to process decimal fractions, for instance in a number magnitude comparison task: (i) different from natural numbers the mere number of digits is not an indicator for the overall magnitude of decimal fractions (e.g., 2.45 is larger than 1.532, although 2.45 is the shorter digit string). (ii) The role of zeros in decimal fractions differs from their role in natural numbers: while zeros to the right of the decimal point with one or more non-zero digits further to the right (e.g., 6.07) do change the value of a decimal fraction, adding one or more zeroes at the right end of a decimal fraction does not change its magnitude (e.g., 6.0 = 6.00, but 60 < 600). Importantly, these differences lead to characteristic errors observed in numerical development. Desmet et al. (2010) investigated misconceptions about decimal fractions in children from grade 3 to 6 using a number magnitude comparison task. Younger children, in particular, tended to overgeneralize their previously acquired knowledge about natural numbers and assumed systematically (but mistakenly) that the more digits a number has, the larger its value. Moreover, children assigned zero the same role as in natural numbers: on the one hand, they implied that adding a zero at the end of a decimal fraction would make it larger. On the other hand, they assumed that adding a zero at the tenths position would not change a decimal fraction’s value.

The processing of decimal fractions in adults has only recently been examined in cognitive psychology. Based on the observation that the numerical distance effect (i.e., faster and less error-prone responses when comparing relatively distant numbers, e.g., 1 vs. 9 as compared to close numbers, e.g., 4 vs. 5, Moyer and Landauer, 1967) did not differ between natural numbers and decimal fractions, Dewolf et al. (2013) concluded that decimal fractions are processed similar to natural numbers. This corroborates the natural number conversion hypothesis (Dewolf et al., 2013) assuming that whenever participants have to compare the magnitude of decimal fractions they might convert the decimal fractions into natural number expressions by simply ignoring the decimal points and then comparing the corresponding natural number.

In contrast to this view, Varma and Karl (2013) suggested that participants do not resort to natural numbers. The authors investigated differences between decimal fraction and natural number processing, proposing two effects other than the distance effect: (i) a syntactic interference effect and (ii) a semantic interference effect. The syntactic interference effect is just another label, introduced by Varma and Karl (2013), for the processing property described by unit-decade compatibility of two-digit numbers (Nuerk et al., 2001). The unit-decade compatibility effect states that magnitude comparisons of unit-decade-compatible natural number pairs (e.g., 42 vs. 57, with 4 < 5 and 2 < 7) are executed faster and less error-prone than comparisons of unit-decade-incompatible pairs (e.g., 47 vs. 62, with 4 < 6, but 7 > 2). Thereby, the unit-decade compatibility effect designates an influence of decision-irrelevant units on the comparison process of the whole numbers. This suggests that the numerical magnitude of a number is represented componentially via the magnitudes of units, 10, 100, etc., complying with the base-10 place-value structure of the Arabic number system (cf. Nuerk et al., 2011 for a review). Varma and Karl (2013) compared this compatibility effect for two-digit numbers with a similar compatibility effect for tenths and hundredths of decimal fractions (e.g., compatible: 0.42 vs. 0.57 and incompatible: 0.47 vs. 0.62). The authors found that the compatibility effect was smaller for two-digit decimal fractions than for two-digit natural numbers and interpreted this finding to imply that different representations are used for decimal fraction and natural number comparison.

Additionally, Varma and Karl (2013) also observed a semantic interference effect: response times for comparisons with congruent numerical magnitude relations between pairs of decimal fractions, respectively natural numbers (e.g., 0.2 vs. 0.53; 0.2 < 0.53 and 2 < 53), were faster than comparisons for incongruent pairs (e.g., 0.23 vs. 0.5; 0.23 < 0.5, but 23 > 5). The authors interpreted this semantic interference effect as a consequence of parallel access to the individual digits, implying that decimal fractions also activate natural number representations in addition to decimal fraction representations. Interestingly, Varma and Karl (2013) compared the semantic interference effect in decimal fractions with the semantic interference effect in natural numbers by converting the decimal fractions into natural numbers by deleting the leading zero and attaching it to the right end of the number (e.g., 0.23 was converted to 23.0). The semantic interference effect was larger in decimal fractions, supporting their hypothesis that decimal fractions are represented differently than natural numbers.

Taken together, recent research seems to suggest that decimal fraction representations are accessed exclusively when comparing decimal fractions with an identical number of digits in the digital string, whereas natural number representations seem to interfere in case of decimal fractions with an unequal number of digits to the right of the decimal point.

In the present study, we will point out that the rejection of the natural number conversion hypothesis by Varma and Karl (2013) might be premature by offering an alternative explanation for their findings of a reduced compatibility effect and an increased semantic interference effect. First, Varma and Karl (2013) suggested other explanations for the smaller compatibility effect for decimal fractions than natural numbers. One of these accounts was that in their experiment the position of the decision-relevant digits was confounded with whether natural numbers or decimal fractions had to be compared with respect to overall number magnitude. Thus, the smaller compatibility effect can also be explained by the difference between natural numbers and decimal fractions regarding the position of the digits decisive for the number magnitude comparison process. For natural numbers the first (leftmost) digit is (primarily) decisive (e.g., in 21 vs. 87, 2 and 8 are relevant), whereas for decimal fractions (<1) the second digit is decisive^2^ (e.g., in 0.21 vs. 0.87, 0 is irrelevant, but 2 and 8 are relevant). Thus, we argue that the important difference between natural numbers and decimal fractions is notational. We suggest that padded natural numbers (like 021 vs. 087) should be processed similarly to decimal numbers (0.21 vs. 0.87) and when leading zeros of decimal numbers are omitted, these should be processed similarly to positive numbers. However, it has not been shown yet that there are similar compatibility effects, when three digit natural numbers – either with differing first digits or with identical first digits but differing second digits – have to be compared (see Nuerk et al., 2011; Klein et al., 2013 for reviews). Hence, we suggest that the observed difference in compatibility effects by Varma and Karl (2013) might be due to different notations used to examine the compatibility effect in natural numbers and decimal fractions.

Second, we also propose an alternative account for the semantic interference effect. In particular, we suggest that the semantic interference effect might alternatively be explained in terms of a congruity effect between the numerical magnitude of the single digits constituting the respective number and the number of digits constituting the digital string. Henceforth referred to as *string length congruity effect*: the comparison of the decisive digits of two decimal fractions can be either congruent (e.g., 2.7 vs. 2.91 with 7 < 9 and 2 vs. 3 digits) or incongruent with the comparison of the string lengths (e.g., 7.14 vs. 7.6 with 1 < 6, but 3 vs. 2 digits). Importantly, first evidence on a somewhat similar effect has already been reported in previous studies. Naparstek and Henik (2010, 2012) and Pansky and Algom (2002) observed that – at least for circular or matrix presentation – the number of digits interfered with the numerical value of the digit. In the numerical value block of Naparstek and Henik (2010), participants had to compare the magnitude of a digit out of a set composed of a varying number of identical digits (e.g., 333) and letter fillers to the standard five. Although the number of digits was irrelevant, participants processed congruent items (number of digits and numerical value being the same: e.g., 333) faster than incongruent items (number of digits and numerical value different: e.g., 3333). Hence, this finding suggests that the number of digits interfered with the processing of the numerical value of a digit. Moreover, in numerical cognition, a common assumption is that numerical and physical magnitudes are not processed independently (e.g., Walsh, 2003; Bueti and Walsh, 2009). Because incongruent items differ from congruent items also in their physical magnitude (i.e., continuous magnitude dimensions such as total surface area, and total “white” color over black background, see Leibovich and Henik, 2013), not only the number of digits, but also their physical magnitude might interfere with processing of the numerical magnitude.

Thus, prolonged reaction times for length incongruent decimal fractions (e.g., 7.14 vs. 7.6 with 1 < 6, but 3 digits vs. 2 digits; Varma and Karl, 2013) might also be explained in terms of interference between the numerical magnitude of the digits constituting the number and the string length of the number. As a consequence, the semantic interference effect observed by Varma and Karl (2013) might be caused by a purely structural difference between natural numbers and decimal fractions, which cannot be matched across item types: as argued above, the mere number of digits is not a valid indicator for the overall magnitude of decimals, whereas it is always a valid indicator for natural numbers. Varma and Karl (2013) tried to account for different visual aspects by adding a zero at the end of natural numbers, but, nevertheless, this difference cannot be controlled for (e.g., 3 < 15 and 3.0 < 15). Therefore, we suggest that the semantic interference effects observed for natural numbers and decimal fractions might have different origins. In the case of natural numbers, numbers with more digits are always larger than numbers with fewer digits, even when the magnitude of the single digits constituting the number with fewer digits is larger than the magnitude of the digits constituting the number with more digits. For instance, when comparing “9 vs. 27,” “27” contains more digits indicating that it is larger than “9.” However, “9” is larger than “2” and “7” and therefore, a componential comparison of 9 with “2” or “7” suggests that “9” should be larger resulting in the proposed semantic interference effect for natural numbers (Varma and Karl, 2013). Thus, for natural numbers, the numerical magnitudes of the single digits interfere with each other. In contrast, in the case of decimal fractions, string length may interfere with numerical magnitude. For instance, when comparing “0.9” with “0.27” and “0.9” is larger than “0.27,” because “9” is larger than “2.” However, the number “0.27” contains more digits than the number “0.9,” which in case of natural numbers would indicate the larger number - and thus, interferes with the decision-relevant comparison of “9” and “2” resulting in the proposed string length congruity effect.

The alternative accounts regarding the reduced compatibility effect for decimal fractions as well as the semantic interference effect observed by Varma and Karl (2013) rely heavily on the notion of componential processing of the individual digits of any multi-digit number as, for instance, indicated by the (unit-decade) compatibility effect (e.g., Nuerk et al., 2001). We want to argue that the processing of decimal fractions may well be integrated into the model of componential number processing. In the present study, we will corroborate this claim by means of a combined empirical and computational modeling approach. In a first step, we will appraise participants’ eye-fixation behavior, while engaged in a number magnitude comparison task involving decimal fractions (e.g., 2.91 vs. 2.43; see also **Table 1** for an overview of different decimal fraction types employed in the present study). This method is used because the registration of eye movements allows for a more fine-grained online evaluation of the comparison process itself. According to the eye-mind hypothesis, the location of eye-fixations and their duration are valid and reliable indicators of what part of a stimulus (i.e., which digit) is processed at a given moment in time and how long this processing lasts (e.g., Rayner and Pollatsek, 1989; Rayner, 1998). In a second step, we aimed at evaluating whether an adapted version of our computational model for multi-digit natural number comparison (Huber et al., 2013a) can also account for the processing of decimal fractions. Thus, the main focus of the present study lies on the computational model, with which we want to show that the findings of Varma and Karl (2013) can be explained by the natural number conversion hypothesis.

**Table 1:** Examples for compatible and incompatible and congruent and incongruent decimal fraction pairs for decimal types a.0c, a.b0, a.bc, and a.b, respectively.

Decimal type	Compatible			Incompatible		
	Numbers	Tenth digit	Hundredth digit	Numbers	Tenth digit	Hundredth digit
a.0c	9.07 vs. 9.39	0 < 3	7 < 9	1.09 vs. 1.51	0 < 5	9 > 1
a.b0	8.10 vs. 8.97	1 < 9	0 < 9	6.54 vs. 6.90	5 < 9	4 > 0
a.bc	4.25 vs. 4.69	2 < 6	5 < 9	3.29 vs. 3.67	2 < 6	9 > 7
	**Congruent**			**Incongruent**		
	**Numbers**	**Tenth digit**	**Number of digits**	**Numbers**	**Tenth digit**	**Number of digits**
a.b	2.7 vs. 2.91	7 < 9	2 vs. 3 digits	7.14 vs. 7.6	1 < 6	3 vs. 2 digits

More specifically, the aim of the eye-tracking experiment was threefold: first, we wanted to explore why the compatibility effect in the study of Varma and Karl (2013) was smaller for decimal fractions than for natural numbers. A recent study by Huber et al. (2013a) showed that in two-digit number comparison the size of the unit-decade compatibility effect increased with the percentage of within-decade filler items (e.g., 43 vs. 47) relative to between-decade critical items. Correspondingly, the authors found that fixations on the irrelevant unit relative to the relevant 10 digits increased, the more within-decade fillers were included. Similarly, the number (and duration) of fixations on irrelevant digits might be indicative of the size of the compatibility effect. Transferring these findings to the case of decimal fraction processing, the smaller compatibility effect for decimal fractions should be associated with fewer fixations on the non-decisive hundredth digits. Such a fixation pattern would indicate that participants process decimal fractions with identical first digits more sequentially than natural numbers with differing first digits. A sequential processing strategy can be identified, when most fixations are on relevant digits and only few fixations on irrelevant digits. Conversely, when participants process digits in parallel, the number of fixations on relevant and irrelevant digits would be more balanced (Moeller et al., 2009). A combination of sequential and parallel processing strategies was already observed for multi-digit number comparison beyond the two-digit number range (Meyerhoff et al., 2012) and therefore, might also be present when comparing decimals. Accordingly, we would expect the tenth digits to be fixated much longer than the hundredth digits as measured by total reading time (TRT; i.e., the time spent fixating a digit).

Second, Varma and Karl (2013) observed that decimal fractions with zeros at the rightmost position (e.g., “0.30”) were responded to faster than other decimals. However, the authors did not investigate whether processing of decimal fractions with zeros directly to the right of the decimal point (e.g., “0.03”) might also be processed faster, providing further support for a privileged role of the digit zero in the comparison of decimal fractions. Similar to faster response times, we would expect that the total fixation time for decimal fractions containing at least one zero will be shorter than for decimal fractions without a zero.

Third, we used the proportion of fixations on tenth and hundredth digits obtained from the empirical study for attentional weighting of the respective digits in the computational model. In the computational model, relevance of the respective digits has to be prespecified in a task demand layer. In principle, two heuristics are possible to obtain suitable values: (i) a trial-and-error approach using starting random values and (ii) an approach using proportions of digits as starting values and adjusting them such that error rates of the simulated data corresponded to the error rates of the empirical data. As we had available proportions of fixations on tenth and hundredth digits from the empirical study, we employed the second approach. Finally, we used reaction times and error rates from the eye-tracking experiment to validate our computational model.

The particular aim of the computational modeling study was to examine whether an extended version of our model for two-digit number comparison (Moeller et al., 2011; Huber et al., 2013c) can account for the findings of the eye-tracking study, which would corroborate the natural number conversion hypothesis for decimal fractions. Therefore, we adapted the existing model to specifically account for the effects observed by Varma and Karl (2013). Most importantly, we extended the network such that three-digit numbers could be compared (as it was already done in the study of Huber et al., 2013b) in order to consider the string lengths of the numbers to be compared. With these measures, a general model of multi-digit number processing encompassing natural as well as decimal fractions can be developed and tested empirically, as done in the current study.

## EMPIRICAL STUDY

### METHODS

#### Participants

Twenty five students of the University of Tuebingen participated in the study (15 female, 10 male) for course credits. Average age was 24.8 years with a standard deviation (SD) of 2.67 years (range 21–33 years). All participants reported normal or corrected-to-normal vision. The study was approved by the local ethics committee of the Medical Faculty of the Eberhard Karls University of Tuebingen. All participants gave their written informed consent.

#### Apparatus

Eye-fixation behavior was recorded by an EyeLink 1000 tracking device (SR-Research, Kanata, ON, Canada). Following 9-point calibration at the start of the experiment as well as drift corrections before each trial, the spatial resolution of the eye-tracking device was less than 0.5 degrees of visual angle at a sampling rate of 1000 Hz. A 20″ monitor set at a resolution of 1024 × 768 pixels and driven at a refresh rate of 120 Hz was used to present stimuli. Viewing distance was about 60 cm. The experiment was programmed using the Experimental Builder software (SR-Research, Kanata, ON, Canada).

#### Stimuli and design

We created 320 pairs of decimal fractions. Decimal fractions were either two- or three-digit numbers ranging from 1.04 to 9.96. Unit digits were identical for number pairs and ranged from 1 to 9. Digits at the tenth and hundredth position ranged from 0 to 9. Participants had to compare four different types of decimal fraction pairs (i.e., 80 decimal fraction pairs for each type). Whereas one of the decimal fractions always consisted of non-zero digits (e.g., 2.91), the other one was generated considering the following constraints: the decimal fraction involved (i) a zero at the tenth position (a.0c; e.g., 2.04), (ii) a zero at the hundredth position (a.b0; e.g., 2.40), (iii) no digit at the hundredth position (a.b; e.g., 2.4), and (iv) no zeros at all (a.bc; e.g., 2.43). Furthermore, we manipulated compatibility and string length congruity (see **Table 1**). To increase the relevance of the hundredth digit, we further included 120 filler number pairs with identical digits at the tenth position (e.g., 7.91 vs. 7.98 or 2.83 vs. 2.8) which resulted in a total of 440 items. Moreover, all digits except for units differed for decimal fraction pairs. Importantly, we balanced overall distance, tenth distance and hundredth distance across all stimulus groups and problem size across all stimulus groups except stimuli with zero at the hundredth position, which had to have a lower overall problem size than the other groups because of its definitional properties.

Stimuli were displayed as white digits on a black background in Courier New (size: 48, style: bold). By using this non-proportional font we ensured that all digits had the same width. X/Y coordinates of decimal fraction pairs were 496/384 and 528/649 pixels or 528/384 and 496/649 pixels, such that leading digits were not presented above each other in order to prevent column-wise processing (see Meyerhoff et al., 2012 for a similar layout). The coordinates of the fixation cross were 512/150 pixels. Decimal fractions consisting of three digits extended to a visual angle of 4.8, horizontally, and 1.2, vertically.

#### Procedure

Participants were assessed individually in a dimly lit room. Instructions requested participants to indicate the larger of two decimal fractions as accurately and fast as possible. When the larger decimal fraction was at the top of the screen, the upper button on a gamepad had to be pressed with the right thumb. Otherwise, when the larger decimal fraction was at the bottom of the screen, the lower button had to press with the left thumb. The position of the larger decimal fraction was counterbalanced for each type of decimal fraction pairs. Each participant completed five practice trials to become familiar with task requirements. Trial order was pseudo-randomized ensuring that the same button was not pressed more than three times in a row. After fixation of the fixation point had been checked by the experimenter, the next item was presented until the participant pressed one of the response buttons, which was immediately followed by the presentation of the fixation point for the next trial.

#### Analysis

Unfortunately, because of a programming error only 320 of the 440 items were presented. These items were randomly drawn from all 440 items such that each participant worked on a different subset of the 440 items. About 85.5 (SD = 3.76) items of the 320 number pairs were filler items (i.e., about 27% like in the original set). Moreover, since the items were a random subset of all items, matching was not affected substantially (see **Table 2**).

**Table 2:** Mean (SD in parentheses) overall distance, tenth distance, hundredth distance, problem size, and number of items for compatible/congruent and incompatible/incongruent decimal fraction pairs for decimal types a.0c, a.b0, a.bc, and a.b, respectively.

Decimal type	Compatibility/congruity	Overall distance	Tenth distance	Hundredth distance	Problem size	Number of items
a.0c	Compatible	0.40 (0.02)	3.66 (0.25)	3.48 (0.15)	10.62 (0.63)	29.56 (2.83)
	Incompatible	0.41 (0.02)	4.42 (0.22)	3.44 (0.26)	10.50 (0.47)	28.76 (2.71)
a.b0	Compatible	0.40 (0.02)	3.69 (0.25)	3.62 (0.15)	11.36 (0.63)	28.60 (2.33)
	Incompatible	0.40 (0.02)	4.37 (0.22)	3.56 (0.26)	10.85 (0.47)	29.76 (1.94)
a.bc	Compatible	0.40 (0.02)	3.67 (0.23)	3.50 (0.23)	11.26 (0.50)	28.56 (2.83)
	Incompatible	0.40 (0.02)	4.39 (0.17)	3.48 (0.14)	10.79 (0.35)	28.88 (2.82)
a.b	Congruent	0.40 (0.02)	3.67 (0.23)	3.77 (0.23)	11.23 (0.50)	30.20 (2.66)
	Incongruent	0.40 (0.02)	4.39 (0.17)	3.69 (0.14)	10.80 (0.35)	30.00 (2.87)

*Please note that participants were presented different numbers of items due to a programming error.*

Three participants exhibited error rates higher than 25% (50% guessing rate) in the string length incongruent condition and were excluded. All subsequent analyses were run on the data of the remaining 22 participants. Additionally, we only included trials followed by a correct answer with response latencies longer than 200 ms and within ±3 standard deviations from the individual mean RT. This trimming procedure resulted in a loss of 6.52% of the data. Only items considered in the RT analysis were also included in the analysis of the eye-fixation data. Moreover, error rates were subjected to an inverse sine transformation before analysis to approximate their binomial distribution with a normal distribution (see e.g., Kirk, 2013, p. 103).

For the analysis of participants’ eye fixation behavior interest areas around each digit and the decimal point were defined with a height of 120 pixels and a width of 39 pixels. We defined the interest areas to be quite narrow, such that the interest areas of each digit and decimal mark were of equal size and interest areas did not overlap. Therefore, it might be possible that participants processed the tenth digit parafoveally while fixating the decimal mark. We exclusively analyzed TRT on the relevant digits (i.e., tenth and hundredth digit) and on critical decimal fractions. Thus, we included only critical decimal fractions with either a zero at the tenth position (e.g., 2.04), a zero at the hundredth place (e.g., 2.40) or only two-digit decimal fractions (e.g., 2.4), respectively, whereas the additional decimal fraction in numbers of the a.bc type was not included (e.g., 2.91), because there is no critical single decimal fraction. For these latter decimal fractions, mean TRTs from both tenth and hundredth digits were calculated.

Reaction times, error rates, and TRT were analyzed by running separate repeated measures analyses of variance (ANOVA). In case of violation of the sphericity assumption for repeated measures ANOVA, the Greenhouse-Geisser (GG) correction was applied to adjust the degrees of freedom. For reasons of readability, the original *df* together with the *GG* coefficient are reported.

### RESULTS

#### Reaction times and error rates

***Processing of zeros and tenth–hundredth compatibility.*** First, we examined, whether we could replicate the relatively smaller compatibility effect found in the study of Varma and Karl (2013) and how zeros in different positions of decimal fractions influenced processing of these decimal fractions. Therefore, we analyzed reaction times and error rates by conducting repeated-measures 3 × 2 ANOVAs with factors decimal type (a.0c, a.b0, and a.bc) and tenth–hundredth compatibility (compatible and incompatible).

**FIGURE 1:**
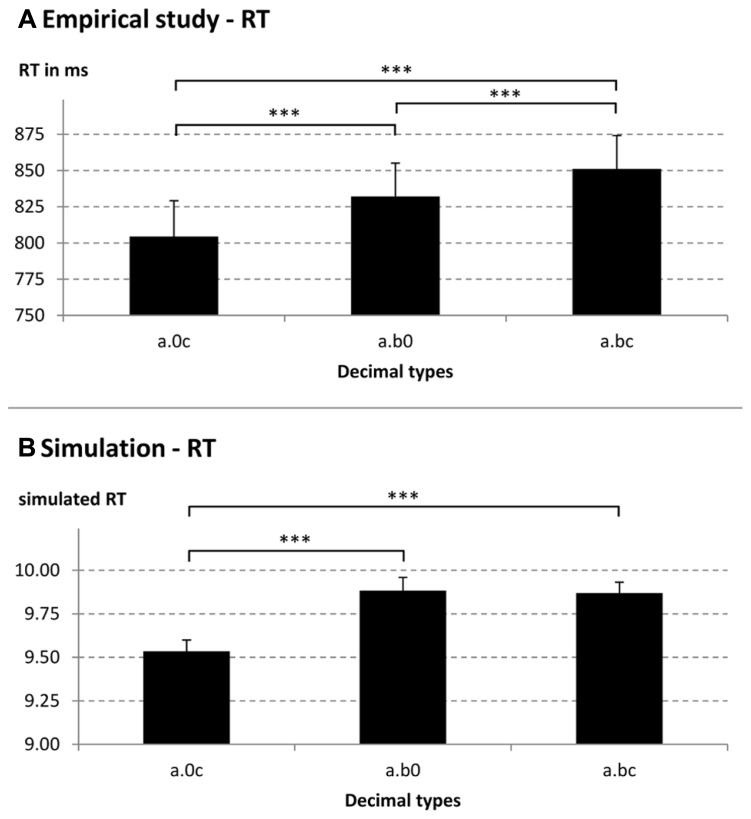
**Reaction times (RT; A, empirical, B, simulated) for different decimal fraction pair types (e.g., a.0c = 2.04 vs. 2.91, a.b0 = 2.40 vs. 2.91, and a.bc = 2.43 vs. 2.91).** ***p < 0.001.

The ANOVA for RT revealed a significant main effect of decimal type [*F*(2,42) = 42.61, *p* < 0.001, $\eta_{p}^{2}$ = 0.67]. Pairwise comparisons indicated that participants compared decimal type a.0c fastest, followed by a.b0 and decimal type a.bc slowest (all *p* < 0.001; a.0c: *M* = 805 ms; a.b0: *M* = 832 ms; a.bc: *M* = 851 ms; see also **Figure 1A**). Thus, we found that zeros facilitated participants’ comparisons of decimal fractions. The main effect of compatibility and the interaction between group and compatibility were not significant, indicating sequential processing of decimal fractions (both *p* > 0.14). Moreover, for error rates we did not find significant main or interaction effects (all *p* > 0.28).

**FIGURE 2:**
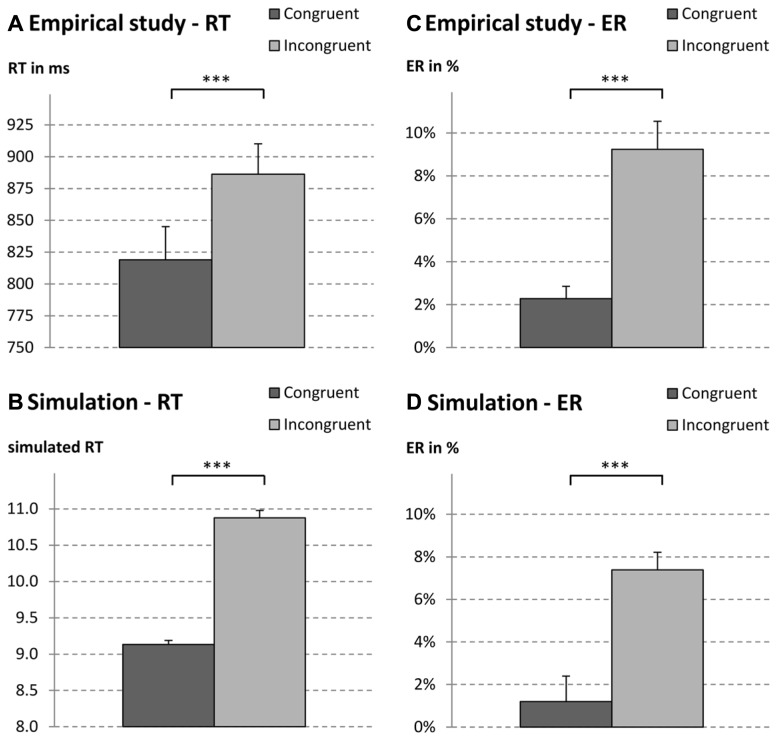
**Reaction times (RT; A, empirical; B, simulated) and error rates (ER; C, empirical; D, simulated) for decimal fraction pair type a.b (e.g., 2.4 vs. 2.91).** The decimal fraction with three digits was either larger (i.e., congruent: 3.98 vs. 3.6) or smaller than the decimal fraction with two digits (i.e., incongruent: 7.63 vs. 7.9). ***p < 0.001.

***String length congruity.*** String length congruity effects on RT and error rates were analyzed by running ANOVAs with congruity as independent variable. Both ANOVAs yielded significant results, indicating shorter RT and lower ER for length congruent than incongruent decimal fraction pairs [RT: *M* = 819 vs. 886 ms; *F*(1,21) = 43.42, *p* < 0.001, $\eta_{p}^{2}$ = 0.67; ER: *M* = 2.29 vs. 9.24%; *F*(1,21) = 34.33, *p* < 0.001, $\eta_{p}^{2}$ = 0.62; see also **Figures 2A,C**].

#### Eye-tracking data

***Processing of zeros and tenth–hundredth compatibility.*** Furthermore, we explored whether participants processed the digits in the decimal fractions sequentially or in parallel. Therefore, we ran a repeated-measures 3 × 2 × 2 ANOVA with factors decimal type (a.0c, a.b0, and a.bc), tenth–hundredth compatibility (compatible and incompatible) as well as digit position (tenth and hundredth digit) and TRT as dependent variable. Results will be reported starting with the three-way interaction, followed by its constituting two-way interactions before main effects will finally be described. Mean TRT for all factors are provided in **Table 3**.

**Table 3:** Mean (SD in parentheses) TRT in ms on tenth and hundredth digits for compatible/congruent and incompatible/incongruent decimal fraction pairs for decimal types a.0c, a.b0, a.bc, and a.b, respectively.

	Tenth digit		Hundredth digit	
Decimal type	Compatible/congruent	Incompatible/incongruent	Compatible/congruent	Incompatible/incongruent
a.0c	181 (42)	178 (40)	32 (28)	34 (27)
a.b0	194 (43)	239 (51)	27 (26)	32 (36)
a.bc	234 (35)	237 (41)	39 (33)	42 (44)
a.b	91 (57)	137 (62)	1 (4)	1 (2)

**FIGURE 3:**
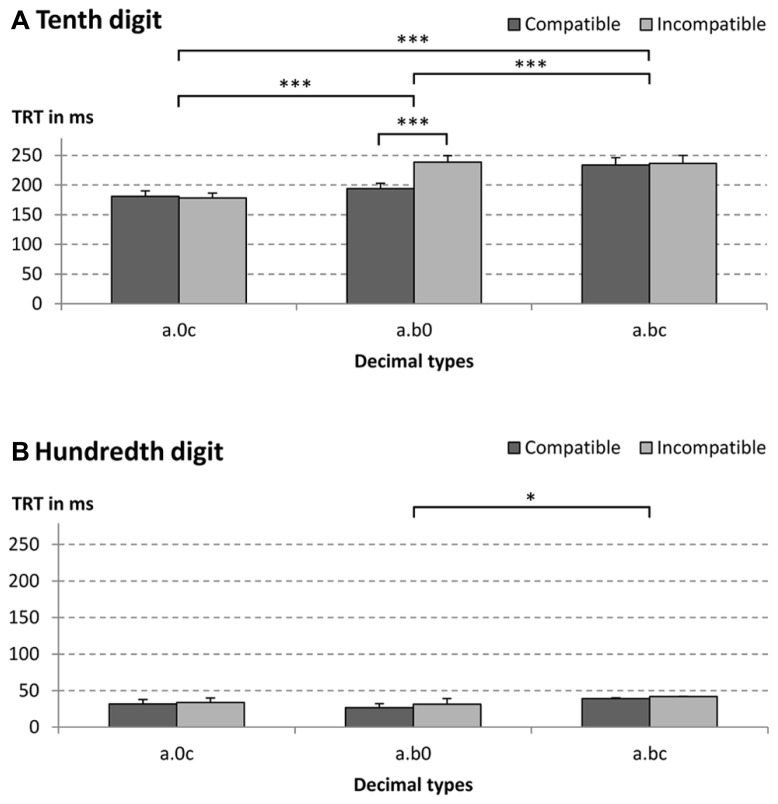
**Mean total reading times (TRT) for (A) tenth and (B) hundredth digit for different decimal fraction pair types (e.g., a.0c = 2.04 vs. 2.91, a.b0 = 2.40 vs. 2.91, and a.bc = 2.43 vs. 2.91).** Decimal fraction pairs were either compatible (e.g., 4.21 vs. 4.67) or incompatible (e.g., 2.45 vs. 2.91). ***p < 0.001, *p < 0.05.

We observed a significant three-way interaction [*F*(2,42) = 5.17, *p* < 0.05, $\eta_{p}^{2}$ = 0.20, *GG* = 0.74; see **Figure 3**]. This interaction was broken down by conducting two 2 × 2 ANOVAs with factors decimal type and tenth–hundredth compatibility separately per digit position (i.e., tenth and hundredth digit).

For the tenth digit (see **Figure 3A**), the interaction between decimal type and tenth–hundredth compatibility was significant, indicating that compatibility effects differed at the tenth digit position [*F*(2,42) = 11.55, *p* < 0.001, $\eta_{p}^{2}$ = 0.36, *GG* = 0.79]. The two-way interaction was further analyzed by conducting (i) two univariate ANOVAs with the factor decimal type for compatible and incompatible items separately and (ii) three univariate ANOVAs with the factor tenth–hundredth compatibility for each decimal type.

For compatible number pairs, we found a significant main effect of decimal type [*F*(2,42) = 38.42, *p* < 0.001, $\eta_{p}^{2}$ = 0.65]. Participants fixated the tenth digits of decimal type a.bc longer than that of other types (both *p* < 0.001), whereas TRT on the tenth digits of decimal types containing a zero did not differ significantly (*p* = 0.38). For incompatible number pairs, the main effect of decimal type was also significant [*F*(2,42) = 32.62, *p* < 0.001, $\eta_{p}^{2}$ = 0.61]. However, different from compatible number pairs, participants fixated the tenth digit of decimal type a.0c shorter than that of other types (both *p* < 0.001), while TRT on the tenth digit of decimal types a.b0 and a.bc did not differ significantly (*p* = 1.00).

Subsequently, we evaluated tenth–hundredth compatibility effects on participants TRT for each of the three decimal types separately. Results indicated that the main effect of tenth–hundredth compatibility was significant for decimal type a.b0 [*F*(1,21) = 22.96, *p* < 0.001, $\eta_{p}^{2}$ = 0.52], but not for the other decimal types (both *F* < 1). While the tenth digits were fixated 45 ms less for compatible than for incompatible a.b0 number pairs, mean compatibility effects for decimal types a.0c and a.bc were -3 and 3 ms, respectively (see also **Figure 3A**).

For the hundredth digit (see **Figure 3B**), the main effect of decimal type was significant [*F*(1,21) = 5.19, *p* < 0.05, $\eta_{p}^{2}$ = 0.20]. Pairwise comparisons revealed that participants fixated the zero of the decimal type a.b0 less than the hundredth digit of decimal types a.bc (*p* < 0.05). All other pairwise comparisons were not significant (all *p* > 0.17). The main effect of tenth–hundredth compatibility and the two-way interaction were not significant (both *F* < 1.24, *p* > 0.54).

Taken together, the three-way interaction revealed a significant compatibility effect on tenth digits for decimal type a.b0. Moreover, we found that zeros were fixated less often than other digits: tenth digits of decimal type a.0c were fixated less than tenth digits of decimal type a.bc in case of compatible number pairs and less than tenth digits of decimal types a.b0 and a.bc in case of incompatible number pairs. Similarly, the zero of decimal type a.b0 (i.e., the hundredth digit) was fixated less than the hundredth digits of other decimal types.

All three two-way interactions were significant [decimal type × compatibility: *F*(2,42) = 18.08, *p* < 0.001, $\eta_{p}^{2}$ = 0.46; decimal type × digit position: *F*(2,42) = 23.06, *p* < 0.001, $\eta_{p}^{2}$ = 0.52; digit position × compatibility: *F*(1,21) = 4.49, *p* < 0.05, *η_p_*^2^ = 0.18]. In the above analyses of the three-way interaction, we already described the interaction between decimal type and tenth–hundredth compatibility for each digit position separately (i.e., for tenth and hundredth digit separately). Moreover, we already analyzed differences between decimal types for each digit positions (as indicated by the two-way interaction between decimal type and digit position). Finally, tenth–hundredth compatibility effects were already discerned for tenth and hundredth digit. Thus, only the analysis of the digit position effects remains to be presented (TRT on tenths - TRT on hundredths) for each decimal type (as indicated by the two-way interaction between decimal type and digit position). First, we analyzed digit position effects for each decimal type separately. Then, we compared digit position effects of decimal types against each other. Generally, the tenth digits were fixated longer than the hundredth digits for all decimal types (all *p* < 0.001) with mean differences between TRT on tenth and hundredth digits for a.0c, a.b0, and a.bc being 147, 187, and 195 ms, respectively. The digit position effect of decimal type a.0c was significantly smaller than the effect of other types (*p* < 0.001), but decimal types a.b0 and a.bc did not differ significantly (*p* = 1.00). Thus, irrespective of decimal type, participants fixated hundredth digits far less than tenth digits indicating an (at least partially) sequential processing strategy.

Finally, all main effects were significant [decimal type: *F*(2,42) = 96.29, *p* < 0.001, $\eta_{p}^{2}$ = 0.82; compatibility: *F*(1,21) = 55.27, *p* < 0.001, $\eta_{p}^{2}$ = 0.73; digit position: *F*(1,21) = 281.66, *p* < 0.001, $\eta_{p}^{2}$ = 0.93]. Comparable to the RT analysis, pairwise comparisons revealed that participants fixated decimal type a.0c shortest, followed by a.b0 and a.bc (all *p* < 0.001; a.0c: *M* = 106 ms; a.b0: *M* = 123 ms; a.bc: *M* = 138 ms). Moreover, the significant compatibility effect indicated shorter TRT for compatible than incompatible number pairs (*M* = 118 vs. 127 ms). Finally, we found longer TRT on relevant tenth digits than on irrelevant hundredth digits (*M* = 210 vs. 34 ms).

***String length congruity.*** Since the critical digit for decimal type a.b did not contain a digit at the hundredth’s position, we analyzed TRT of tenth digits separately by conduction a paired *t*-test. Thereby, we evaluated whether string length congruity also affected participants’ fixation pattern in addition to response times. The paired *t*-test for the string length congruity effect of decimal type a.b revealed a significant congruity effect. Participants fixated the tenth digits of incongruent number pairs longer than those of congruent number pairs [congruent: *M* = 91 ms vs. incongruent *M* = 137 ms; *F*(1,21) = 40.06, *p* < 0.001, $\eta_{p}^{2}$ = 0.66].

## SIMULATION

### MODEL FOR TWO-DIGIT NUMBER COMPARISON

**FIGURE 4:**
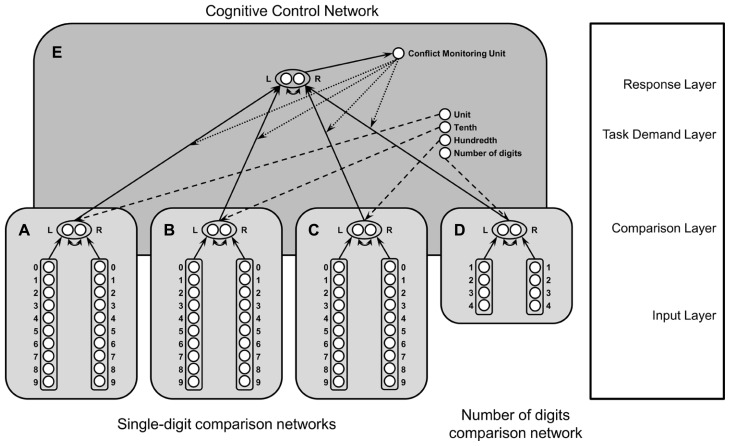
**Architecture of the neural network model for decimal fraction processing: networks (A) to (C) depict digit comparison networks for (A) units, (B) tenth digits and (C) hundredth digits, and **(D)** the number of digits comparison network.** These networks are integrated into the neural network of cognitive control **(E)** as suggested by Verguts and Notebaert (2008). L, left digit larger node; R, right digit larger node.

To simulate the processing of decimal fractions, we adapted the computational model of Huber et al. (2013c), which simulates the comparison of two-digit numbers using an artificial neural network (see **Figure 4**). This model consists of two single-digit comparison networks for tens and units and a cognitive control network, which was inspired by the cognitive control network of Verguts and Notebaert (2008).

The single-digit comparison networks are composed of an input layer and a comparison layer. In the input layer the representation of digits is modeled using a place coding system with a fixed Gaussian distribution for each digit (see Verguts et al., 2005, for a similar approach). Thus, for each digit there is a unit which is activated most and units coding digits of a similar magnitude are activated to a lesser degree depending on their numerical distance to the respective digit [i.e., f (i,j) = exp(-10 * |i - (j + 1)|) for node *i* and digit *j*]. The units of the input layer are connected via forward connections to two comparison nodes, one coding “left digit larger” and the other one coding “right digit larger.” The activation of comparison nodes is calculated by the weighted sum of input nodes reduced by inhibitory connections between comparison nodes with weights *w^inh^* = -2. The activation function of the comparison nodes is a sigmoid function.

Before single-digit comparison networks were integrated into the cognitive control network, they were trained using the delta rule (Widrow and Hoff, 1960). To do so, weights between input and comparison layer were initialized by generating pseudo-random values in the interval [-1; 1]. The training comprised 100,000 trials, after which the network compared all combinations of single-digit number pairs correctly. Only one network was trained. Connection weights were reused in the second network.

In the cognitive control network, activity of input nodes is propagated to the comparison nodes following formula A1 of Verguts and Notebaert (2008). However, instead of an indicator function we use the activation propagated from the input layer to the comparison layer because in our model the input of the cognitive control network is the output of the single-digit comparison networks. Hence, instead of using color information like in a Stroop task (see Verguts and Notebaert, 2008), we use the comparison information of one of the digits being larger. This information is not prespecified in the input layer (as it would be when using the original network architecture of Verguts and Notebaert, 2008), but is generated by the single-digit comparison networks. Comparison nodes are connected to two response nodes via forward connections. Again, one node codes “left digit larger” and the other one codes “right digit larger” and response nodes are connected to each other via inhibitory connections with *w^inh^* = -0.5. Moreover, activation in the comparison layer is modulated by task nodes for the two tasks of comparing either tens or units, as described by equation A2 in Verguts and Notebaert (2008).

Effects of cognitive control are simulated via a conflict monitoring unit. The degree of conflict detected by the conflict monitoring unit is used to adapt the connection weights between task demand nodes and corresponding comparison nodes according to the Hebbian learning rule in equation A3 of Verguts and Notebaert (2008).

Response times are simulated by counting the number of steps needed until one of the comparison nodes reaches a fixed threshold θ of 0.8. Since it is possible that this threshold is never reached, the maximum value for simulated response times was set to 200.

### MODIFICATIONS OF THE EXTENDED MODEL FOR DECIMAL FRACTIONS

The model for two-digit numbers could be extended to simulate decimal fraction comparison without introducing further qualitative changes to the original model structure (see **Figure 4**). Since decimal fractions consisted of up to three digits, we simply added another single digit comparison network. Moreover, to simulate the comparison of numbers comprising different numbers of digits (i.e., two vs. three digits), we added another network for the comparison of the number of digits, which was very similar to the single-digit comparison networks (see **Figure 4C**). The comparison of the number of digits can be simulated by the very same network architecture as the comparison of digits (e.g., Verguts and Fias, 2004; Santens and Verguts, 2011). However, tuning curves have to be broader for non-symbolic comparisons (i.e., number of digits) than symbolic comparisons (i.e., comparison of numerical magnitude; Santens and Verguts, 2011) using the following activation function: f (i,j) = exp(-|i - (j + 1)|) for node *i* and number of digits *j*. Moreover, the number of nodes was reduced to four nodes for the comparison of numbers with up to four digits (see **Figure 4D**). Four digits were chosen to extend the network easily for comparisons of numbers with up to four digits, but also three or five or more digits would have been feasible. We also had to extend the task demand layer. Differing from the two-digit comparison network, there are nodes for the comparison of units, tenths, hundredths, and the number of digits.

Furthermore, activation of task nodes and connection weights between comparison layer and response layer were modified to simulate different attentional weighting of the respective digits of decimal fractions. Attentional weighting values were inspired by the relative frequency of fixations on tenth and hundredth digits. Activity of the task node for units was set to 0 as a negligible number of fixations fell on unit digits, namely 2.5% of all fixations. The task node for the tenth digits was set to the largest value of 1.5, because they were fixated most (i.e., 84% of all fixations). To implement the very weak interference of hundredth digits, with only 13% of all fixations on hundredth digits, activation was set to the very low value of 0.01. A similar pattern of values was chosen for the connection weights between comparison layer and response layer: 0.1, 1.0, 0.1, for unit, tenth and hundredth digits, respectively. A different weighting of relevant and irrelevant tasks was also suggested by Santens and Verguts (2011). In their computational model the representational layer of the irrelevant dimension was multiplied by a parameter Θ with the value of 0.15, which determined the size of the size congruity effect (see also Schwarz and Ischebeck, 2003).

Moreover, to capture the increased impact of zero in decimal fraction processing (Varma and Karl, 2013), single-digit comparison networks were not trained using a distribution obtained from a Google-survey (see also Verguts and Fias, 2006; Moeller et al., 2011; Huber et al., 2013b). Instead, the frequency of occurrence of 0 in the training set of single-digit numbers was 15% larger and the frequency of occurrence of 1 was 5% larger than the frequency of occurrences of the other digits. By increasing the frequency of occurrence of 0, comparison with 0 will be trained more often, thereby; increasing corresponding weights between input nodes and comparison nodes, thus leading to faster comparisons with zero (see also Verguts et al., in press, for frequency effects). We also tried only to increase the frequency of 0, but this was not as effective as additionally increasing the frequency of 1. However, other values might also have resulted in a similar effect. Moreover, we slightly increased the training phase to 120,000 trials (in contrast to 100,000 trials in Huber et al., 2013c).

Attentional weights for the comparison of the number of digits were set to slightly lower values than the attentional weights for the comparison of tenth digits (i.e., activation of task node: 1.0 and connection weight between comparison and response layer: 0.7). However, each comparison of the number of digits was trained more often than the comparison of single digits in the single-digit comparison networks (i.e., 100,000 trials for 4 nodes vs. 120,000 trials for 10 nodes of the other single-digit comparison networks). Thereby, the comparison of number of digits is faster than the magnitude comparison of digits, resulting in a more pronounced string length congruity effect.

Other parameters of the cognitive control network are mostly identical to the ones used by Huber et al. (2013c) and Verguts and Notebaert (2008): *τ* = 0.8, β_in_ = 0.2, *w^inh^* = -0.5, *C* = 0.7, β_con_ = 1, λ_con_ = 0.8, λ*_w_* = 0.7, α*_w_* = 1, and β*_w_* = 0.5.

### PROCEDURE AND ANALYSIS

The same stimuli as in the empirical study were used. However, artificial networks were presented with the entire set of 440 items^3^. We simulated 22 participants by creating 22 randomizations of trial orders. Since we added random Gaussian noise at each time step (*M* = 0, *SD* = 0.11), simulated RT and error rates were different for each simulated participant. Similar to the empirical study, we excluded simulated RT of trials which were not solved correctly from further analyses resulting in a loss of 2.6% of the data. Moreover, error rates were subjected to the inverse sine transformation prior to analyses to approximate a normal distribution.

### RESULTS

#### Processing of zeros and tenth–hundredth compatibility

To examine the tenth–hundredth compatibility effect and how zeros influence processing of decimal fractions, we analyzed simulated RT and error rates by conducting two repeated-measures 3 × 2 ANOVAs with factors decimal type (a.0c vs. a.b0 vs. a.bc) and tenth–hundredth compatibility (compatible vs. incompatible). For RT data, the main effect of decimal type was significant [decimal type: *F*(2,42) = 18.03, *p* < 0.001, $\eta_{p}^{2}$ = 0.46]. Mean RT for a.0c, a.b0, and a.bc were: 9.53, 9.88, and 9.87, respectively. Similar to the findings for the behavioral RT, we found that decimal type a.0c was compared faster than the other decimal types (all *p* < 0.001). However, different from the empirical RT findings, decimal type a.b0 did not differ significantly from decimal type a.bc (*p* = 1.00, corrected for multiple comparisons). The main effect of tenth–hundredth compatibility and the interaction between decimal type and tenth–hundredth compatibility were not significant (both *p* > 0.24). Moreover, in line with the empirical findings, we did not find any significant main or interaction effects for error rates (all *p* > 0.05). Thus, as indicated in **Figure 1**, simulated RT replicated the finding of faster RT for decimal type a.0c compared to the other decimal types studied. However, the model could not account for faster RT for decimal type a.b0 compared to decimal type a.bc.

#### String length congruity

String length congruity effects for simulated RT and error rates were analysed by running two paired *t*-tests. Both *t*-tests were significant indicating shorter simulated RT and lower simulated ER for length congruent than incongruent decimal fraction pairs [RT: *M* = 9.14 vs. 10.87; *F*(1,21) = 265.43, *p* < 0.001, $\eta_{p}^{2}$ = 0.93; ER: *M* = 1.02 vs. 7.05%; *F*(1,21) = 65.98, *p* < 0.001, $\eta_{p}^{2}$ = 0.76]. Thus, as depicted in **Figure 2** (for RT see **Figures 2A,B** and for ER see **Figures 2C,D**), simulated RT as well as ER were in accordance with the empirical findings.

## DISCUSSION

In the present study, we aimed at providing an alternative explanation for the findings of Varma and Karl (2013), who had reported a smaller compatibility effect and a larger semantic interference effect for decimal fractions as compared to natural numbers. These two findings would be consistent with the hypothesis that decimal fractions are represented differently and, thus, decimal fractions would be processed differently when compared to natural numbers. Therefore, we examined whether rejecting the natural number conversion hypothesis, stating that decimal fractions are processed similar to natural numbers, may have been premature. Our results indicated that both findings can also be explained by relying on componential processing of multi-digit natural numbers in line with the natural number conversion hypothesis (see also Dewolf et al., 2013). It provides a more parsimonious explanation for findings in decimal fraction comparison, because additional decimal fraction representations do not have to be assumed in order to explain how participants compare decimal fractions. Thus, the present study supports the notion that natural numbers and decimal fractions are processed similarly.

### COMPATIBILITY EFFECT

We did not find a significant compatibility effect for reaction times or error rates data in our behavioral experiment. This result suggests that hundredth digits interfere less, when comparing decimal fractions than when comparing natural numbers with respect to their magnitude. At first glance, this pattern of results is in line with the interpretation of Varma and Karl (2013), suggesting distinct representations for decimal fractions. However, there is another explanation for the absence of the compatibility effect in our study: participants may have compared the decimal fractions – at least in part – sequentially (e.g., Moeller et al., 2009; Meyerhoff et al., 2012). This assumption could be tested systematically in the present study by evaluating participants’ eye fixation behavior. In particular, Moeller et al. (2009) hypothesized that a sequential comparison of two two-digit numbers would primarily lead to fixations on tens and to only a very small number of fixations on units. Moreover, fixations should not differ between compatible and incompatible number pairs. Analogously, sequential processing of decimal fractions in our study would result in long TRT on the tenth digits and only very short TRT on the hundredth digits. In fact, we actually found that pattern for number pairs including no zeros (i.e., a.bc) or zero at the tenth position (i.e., a.0c). There was no compatibility effect for hundredth digits, and TRT on tenth digits were about six times longer (ranging from 1.6 to 223 times for individual participants) than TRT on hundredth digits. In order for (automatic) processing of the hundredth digits to interfere sufficiently with the processing of tenth digits (i.e., to elicit a significant compatibility effect), participants have to fixate the hundredth digits to a certain extent (cf. Huber et al., 2013a). Thus, our findings indicate that hundredth digits could be ignored more easily, when comparing decimal fractions with identical unit digits. The most probable reason for this is that participants processed decimal fractions sequentially and not in parallel, which is typical for two-digit numbers.

Nevertheless, we found a significant compatibility effect for TRT on the tenth digits of decimal fractions having zero at the hundredth position (i.e., a.b0). Thus, whereas compatibility did not affect participants’ reaction times and error rates, it modulated participants’ fixations on the tenth digits. Therefore, zero at the hundredth position might mislead participants to assume that this decimal fraction is smaller than the other one. As a consequence, participants had to fixate the tenth digit longer in incompatible than in compatible decimal fraction pairs to overcome this bias.

Finally, also in our computational simulations we did not find a significant compatibility effect, although we simulated the comparison of decimal fractions, using a fully componential model. As outlined above, this finding is obviously a consequence of the very low attentional weighting of the hundredth digits, eliminating the compatibility effect. Increasing the activity of the task demand nodes and the weights of the connection between task demand nodes and comparison layer for the hundredth digits would result in reliable compatibility effects for all decimal fraction types. Similarly, we would predict that increasing the relevance of the hundredth digit in an empirical study would result in a reliable compatibility effect. One way to achieve this would be to increase the number of filler items. Macizo and Herrera (2011; see also Huber et al., 2013a) found that the size of the unit-decade compatibility effect in two-digit numbers depends on the number of filler items: the more within-decade filler items, the larger the compatibility effect. Therefore, it is possible that increasing the number of filler items would have led to a significant compatibility effect. To conclude, also our computational modeling suggested that a smaller compatibility effect or even the absence of a compatibility effect for decimal fractions does not necessarily imply a distinct representation of decimal fractions.

### STRING LENGTH CONGRUITY EFFECT

Similar to the results of Varma and Karl (2013), string length congruity had a very strong impact on the comparison of decimal fractions. In particular, we found reliably longer reaction times and higher error rates for length incongruent than length congruent decimal fraction pairs (i.e. congruent: 2.7 vs. 2.91 with 7 < 9 and 1 vs. 2 digits; incongruent: 7.14 vs. 7.6 with 1 < 6, but 2 vs. 1 digits). We even had to exclude three participants from the analysis because of their very high error rates (almost at chance level) when comparing string length incongruent items. A possible explanation may be that these three participants confused, for instance, 2.06 with 2.60. However, if so, they made this error not systematically. Otherwise, we would have observed a more systematic error pattern and error rates close to 100% for incongruent pairs, which was not the case. Instead, error rates were close to chance level (i.e., 50%). Moreover, our participants were university students who not only should have learnt the decimal notation in school, but are also confronted with it in their statistics courses. Therefore, we are confident that they should at least have had a basic understanding of decimal number notation. Nevertheless, a further study would be required to investigate whether a poor understanding of decimal number notation might explain the poor performance of some students when comparing decimals.

Varma and Karl (2013) suggested that the string length congruity effect is caused by semantic interference of natural and decimal fraction mental representations. However, our simulation provides an alternative explanation. We did not include specific representations for decimal fractions in our network architecture. Instead, we added representations for the number of digits of decimal fractions. Thereby, we were able to simulate the observation of longer reaction times for incongruent than for congruent decimal fraction pairs (i.e., the string length congruity effect). The string length congruity effect may thus be just another example for the assumption that numerical magnitude and physical magnitude (as reflected by the number of digits) are not processed independently (Pansky and Algom, 2002; Naparstek and Henik, 2010, 2012). Numbers with more digits also have a larger physical (i.e., horizontal) extension. Thus, continuous magnitude dimensions (e.g., total surface area, and total “white” color over black background) might interfere with the processing of numerical magnitudes (see also Leibovich and Henik, 2013, for a similar suggestion regarding numerosities). This notion is further supported by the fact that we used a very similar network architecture as in the study of Santens and Verguts (2011), who simulated the size congruity effect (Henik and Tzelgov, 1982) using a dual route model with separate representations for numerical magnitude and physical magnitude. In their model the shared decision account was implemented, according to which the interaction between comparison of numerical and physical magnitude takes place at the decision level (e.g., Schwarz and Heinze, 1998; Santens and Verguts, 2011). Contrarily, the shared representation account suggests that numerical and physical magnitude share the same representation (e.g., Walsh, 2003; Bueti and Walsh, 2009). We adapted the approach of Santens and Verguts (2011) by creating separate representations for numerical magnitude and number of digits, the latter implemented as a discrete measure of horizontal extent. Thereby, the network architecture of the present study was motivated by the shared decision account for the interaction of numerical magnitude and continuous physical magnitude as proposed by Santens and Verguts (2011). Hence, we suggest that not only numerical and continuous physical magnitude may interfere at the decision level (as for the size congruity effect), but also numerical magnitude and the discrete number of digits.

Taken together, our simulation study indicates that the number of digits interferes with the comparison of numerical magnitudes (as measured by the string length congruity effect). However, this influence of the number of digits might indicate an influence of physical magnitude (i.e., vertical extension) on the processing of number magnitude. Further studies are needed to disentangle these two possible origins of the string length congruity effect.

### ROLE OF ZERO

Interestingly, we observed that decimal fractions with a zero at the tenth position were processed fastest. Importantly, however, we were only able to simulate this finding by increasing the frequency of occurrence of zeros in single-digit number comparison. Yet, this modification did not explain why decimal fractions with ending zeros were compared faster than decimal fractions without zeros. Varma and Karl (2013) suggested that zeros may be privileged in cognitive processing. Our simulation, implementing such a privileged processing of zeros, suggested that this privileged processing affected decimal fractions with zeros at the tenth digit position, but not those with zeros at the hundredth position, which was more or less neglected by the participants in our study and therefore, should not have affected response times. However, an alternative account might be that participants did not process decimal fractions with zeros at the end componentially. Instead, they might have processed the fractional part of the decimal fraction holistically. For instance, when comparing 2.91 and 2.40, participants ignored the unit digits and compared 91 and 40 instead. Specific processing advantages (as indicated be faster response times) for multiples of ten have already been reported before (Brysbaert, 1995; Nuerk et al., 2002). Thus, faster responses for decimal fractions with zeros at the end might not necessarily indicate a privileged role of zeros but a privileged role of multiples of ten, which a model of strictly componential processing cannot account for.

### PERSPECTIVES

By assuming that processing of decimal fractions is not different from processing of natural numbers, our computational model also allows for predictions about the processing of natural numbers and decimal fractions within one model framework. In accordance with our findings for decimal fractions, we would expect that the unit-decade compatibility effect is smaller for three-digit numbers with identical hundred digits as compared to the hundred-decade compatibility effect for three-digit numbers.

Necessarily, the natural number conversion hypothesis and the model architecture employed in the present study suggest that the number of digits should influence the comparison of numbers not only when comparing decimal fractions, but also when comparing two natural numbers. However, differing from decimal fractions in natural numbers, the number of digits is always congruent with numerical magnitude. Therefore, different numbers of digits can only facilitate the comparison of two natural numbers, but never interfere with the comparison of two natural numbers. Nevertheless, the computational model architecture predicts that natural numbers containing different numbers of digits should be compared faster than natural numbers with the same number of digits, even if distance and problem size are matched.

Moreover, the computational model, which served as a basis for the extended network presented in the current study, was developed to simulate effects of cognitive control observed in two-digit number processing. It was able to account for the proportion congruity effect found by Macizo and Herrera (2013) and predicted a Gratton effect (Gratton et al., 1992) in two-digit number comparison. Hence, the computational model predicts that also the comparison of decimal fractions should be under cognitive control modulating the relevance of tenth and hundredth digits. Regarding the tenth–hundredth compatibility effect, we would expect it to be more pronounced in a stimulus set with a smaller proportion of incompatible relative to compatible number pairs, as found by Macizo and Herrera (2013) for the case of two-digit numbers. Moreover, in the original computational model (Huber et al., 2013c) cognitive control was implemented to act locally on a trial-by-trial basis and, thereby, it was able to simulate the Gratton effect in two-digit number comparison. Transferred to the case of decimals, the model thus predicts that participants should adapt to different proportions of incompatible trials when comparing decimal fractions on a trial-by-trial basis as well. However, the computational model suggests that not only the relevance of digits should be influenced by processes of cognitive control, but also the inferential influence of the number of digits. This means that the inferential influence of the number of digits should depend on the proportion of string length incongruent to congruent number pairs (in the sense of a proportion congruity effect). In the current computational model, the network for comparing different numbers of digits was added in the same way as the network for the magnitude comparison of digits. Therefore, the same processes of cognitive control, which modulate the relevance of tenth and hundredth digits, should also modulate the influence of the number of digits. In a similar vein as for the relevance of tenth and hundredth digits, the computational model predicts that the influence of the number of digits should be smaller for higher proportions of incongruent trials. More specifically, the string length congruity effect should be larger in a condition with only 25% incongruent trials than in a condition with 75% incongruent trials (see Macizo and Herrera, 2013, for a similar proportion congruity manipulation on the unit-decade compatibility effect). Moreover, as cognitive control was implemented to act locally on a trial-by-trial basis, the computational model also predicts a Gratton effect for the string length congruity. These predictions are a direct result of the network architecture employed to simulate the string length congruity effect and have to be demonstrated in future empirical studies.

Furthermore, we simulated the processing of decimal fractions using a fully componential model. With this architecture, however, the model was not able to account for the observed faster responses for decimal fractions with zero at the rightmost position. We suggested that the fractional part of these decimal fractions might be processed holistically because of its similarity to multiples of ten (e.g., the fractional part of 2.40 is 40). In the study of Moeller et al. (2011), the fully componential model was favored, because it was more parsimonious than the hybrid model assuming that there exit both a componential as well as a holistic representation of two-digit numbers (e.g., Nuerk and Willmes, 2005). However, the present study suggests that at least some two-digit numbers (i.e., whole 10) might be processed holistically favoring a hybrid model of two-digit number processing.

## CONCLUSION

The present study aimed at investigating the processing of decimal fractions. Currently, there is a debate on whether decimal fractions are processed like natural numbers (natural number conversion hypothesis) or whether there exist mental representations of decimal fractions, which are distinct from those of natural numbers. The latter suggestion of distinct representations for decimal fractions was supported (i) by the finding of a smaller compatibility effect in decimal fraction than in natural number comparison and (ii) by a semantic interference effect indicating that natural number representations interfere with the comparison of decimal fraction representations. In the present study, we investigated whether these differences indeed indicate that decimal fractions are processed differently from natural numbers. To do so, we provided another account for the semantic interference effect. We proposed that a string length congruity effect evoked by an incongruity between comparison of the magnitude of digits and the physical length could also account for the semantic interference effect. To evaluate this suggestion, we conducted an eye-tracking study and simulated the empirical findings using a computational model. Importantly, in the computational model we did not implement specific decimal fraction representations. Instead, our model was an extension of our fully componential model for two-digit number comparison. To account for the proposed string length congruity effect, we added a network for the comparison of the number of digits. The computational model could account for the smaller compatibility effect in decimal fraction comparison and for the string length congruity effect providing further support for the natural number conversion hypothesis.

## Conflict of Interest Statement

The authors declare that the research was conducted in the absence of any commercial or financial relationships that could be construed as a potential conflict of interest.

## Footnotes

1 Please note that different cultures use different symbols for the decimal mark. The two most common decimal marks are a dot “.” and a comma “,”.

2 Please note that all decimal fractions in the study of Varma and Karl (2013) were smaller than 1 and had 0 as the leading digit.

3 Please note that results were comparable when analyzing only a random subset of 320 items of the 440 items corresponding to 320 items which were presented to the participants in the empirical study.
